# Inhibiting Caspase-12 Mediated Inflammasome Activation protects against Oxygen-Glucose Deprivation Injury in Primary Astrocytes

**DOI:** 10.7150/ijms.44330

**Published:** 2020-07-19

**Authors:** Lu Liu, Manli Chen, Kun Lin, Xuwu Xiang, Yueying Zheng, Shengmei Zhu

**Affiliations:** Department of Anesthesiology, The First Affiliated Hospital, College of Medicine, Zhejiang University, 79 Qingchun Road, Hangzhou 310003, People's Republic of China.

**Keywords:** stroke, ischemia reperfusion injury, astrocytes, caspase-12, NLRP3-inflmmasome

## Abstract

Stroke is one of the leading causes of death worldwide. Accumulating evidence suggests that NLRP3 inflammasome activation plays an important role in ischemic stroke injury. However, the existence of the NLRP3 inflammasome in astrocytes remains controversial. In this study, we demonstrated the presence of the NLRP3 inflammasome in primary mouse astrocytes and investigated the role of caspase-12 in NLRP3 inflammasome activation and cell injury in an *in vitro* astrocyte oxygen-glucose deprivation (OGD) model. Astrocytes exposed to 2, 3, and 4 h of OGD exhibited increased cell injury and apoptosis, and the protein levels of caspase-12, cleaved caspase-3, NLRP3 inflammasome components, and IL-1β were also significantly elevated. Interestingly, pretreatment with the caspase-12-specific inhibitor Z-ATAD-FMK attenuated cell injury and apoptosis and decreased the levels of NLRP3, caspase-1, IL-1β, and cleaved caspase-3 in the OGD group. In conclusion, Z-ATAD-FMK protected astrocytes against OGD-induced cell death and inhibited NLPR3-inflammasome activation. Our results indicate that caspase-12 and its potential regulation of NLRP3 inflammasome activation might be a promising target for treatment of ischemic stroke.

## Introduction

Ischemic stroke is one of the leading causes of death and serious long-term disability with prolonged physical, emotional, social, and financial consequences. A total of 113 million disability-adjusted life years (DALYs) are due to stroke; moreover, 10.3 million new strokes occur every year, of which 58% of DALYs and 67% of new cases are ischemic strokes 1. There are several therapeutic options for ischemic stroke, including thrombolytic therapy, intravenous alteplase, intravascular thrombectomy, and neuroprotective drugs. However, the effectiveness of these treatments is limited in clinical practice due to narrow therapeutic windows 2. Therefore, alternative effective medical interventions are urgently needed.

Astrocytes, as major glial cells in the central nervous system (CNS), play an important role in maintaining normal neuronal function, including regulation of cerebral blood flow, extracellular fluid, ion homeostasis, ion transportation, energy supply, synaptic function, and synaptic remodeling 3. In ischemic stroke, accumulating evidence suggests that astrocytes play fundamental roles in the pathogenesis of ischemic neuronal death; however, the detailed mechanisms remain unclear. Importantly, endoplasmic reticulum stress (ERS) is particularly damaging to ischemic brains and was found to play a role in the pathophysiology of hypoxia/ischemia-induced neuronal death 4. ERS can initiate the activation of caspase-12. Caspase-12 activation and processing have been detected following ischemic stroke, but the role of caspase-12 in apoptosis and inflammation is controversial. Experiments have shown that caspase-12 is responsible for further activation of caspase-3 and ERS-induced cell apoptosis in astrocytes following ischemic stroke 5-7. Caspase-12 is classified in the group I family of caspases, which are characterized as inflammatory caspases 8, 9. Caspase-12 has been shown to induce apoptosis in different cell and animal models; extensive cellular crosstalk may be involved 10-12. However, there is limited evidence of other downstream mechanisms of caspase-12 in astrocytes subjected to ischemia/reperfusion (I/R) injury. Studies have focused on the relationship between caspase-12 and inflammation in an effort to understand the underlying mechanism. At present, it is believed that caspase-12 negatively modulates the innate inflammatory response as well as inflammasome activation and caspase-1 activity 8, 13. However, Saleh et al. have challenged this opinion, as they failed to see increased caspase-1 activation in caspase-12-deficient mice in a recent study 14. Moreover, irremediable ERS after I/R injury is associated with nucleotide-binding domain leucine-rich repeat containing receptor pyrin domain containing 3 (NLRP3) inflammasome activation 15. The NLRP3 inflammasome has been reported to be involved in the pathophysiology of ischemic stroke 16. Nevertheless, the existence of the NLRP3 inflammasome in primary mouse astrocytes remains controversial 17, 18.

To date, the relationship between caspase-12 and the NLRP3 inflammasome in ischemic stroke is unclear. The aims of this study were to investigate the existence of the NLRP3 inflammasome in primary mouse astrocytes, as well as the effect of caspase-12 on apoptosis, and to explore the molecular mechanisms of the caspase-12/NLRP3 inflammasome pathway.

## Materials and Methods

### Primary mouse astrocyte cultures

Primary astrocytes were separated from cerebral cortices of postnatal (0-1 day old) male and female C57BL/6 mice, as described previously 19, 20. The astrocytes were cultured in complete medium composed of high-glucose Dulbecco's modified Eagle medium (DMEM; 4.5 g/l D-glucose, HyClone) supplemented with 10% fetal bovine serum (BI, #04-001-1A) and 1% penicillin/streptomycin. Cells were plated in T-75 flasks (Costar; Corning) at a density of three cortices per flask precoated with poly-D-lysine (10 µg/ml; Sigma-Aldrich). The cells were grown for at least 7 days at 37°C with 5% CO_2_, and the medium was changed every other day. After 8-10 days, confluent astrocytes were purified of microglia by shaking for 12-16 h at 250 rpm at 37°C on an orbital shaker followed by culture in medium containing 20 µM cytosine-1-β-D-arabinofuranosid (Sigma-Aldrich) for 2-3 days. Dissociated cells were then re-plated at a density of (0.03-0.05) × 10^6^/cm^2^ in 6-well plates precoated with poly-D-lysine and then cu ltured at 37°C in a humidified incubator with 5% CO_2_. After 5-7 days, cultures at 90-95% confluence were used for subsequent experiments. Immunofluorescence (GFAP, 1:100, Cell Signaling Technology, #12389; IBA-1, 1:100, Novus, #NBP2-19019) and western blotting were used to test the purity of the astrocytes, which was found to be more than 95%.

### Oxygen glucose deprivation and reperfusion

Oxygen-glucose deprivation (OGD) experiments were performed using a humidified incubator chamber at 37 °C with 95% nitrogen and 5% CO_2_, and the culture medium was replaced with DMEM without glucose. After 1-4 h of OGD, cultures were removed from the chamber and reperfused for 24 h with complete medium in a regular incubator. Control cell cultures were incubated in high-glucose DMEM under normoxia for the corresponding duration. Cell viability was measured using the lactate dehydrogenase (LDH) cytotoxicity assay.

### Inhibition of caspase-12-mediated apoptosis using Z-ATAD-FMK

To verify if I/R-induced cell injury and apoptosis are mediated by caspase-12, primary mouse astrocytes were pretreated with the caspase-12 inhibitor Z-ATAD-FMK (ZAF; R&D; #FMK013). Briefly, primary mouse astrocytes were preincubated with 0, 0.05, 0.5, 5, or 10 μM ZAF 24 h before OGD. Then, the supernatant was replaced with normoxic medium, and the cells were incubated for another 30 min before OGD.

### Lactate dehydrogenase cytotoxicity assay

The LDH detection kit is a colorimetric assay used to quantify cell death and cell lysis based on the release of LDH from the cytosol of damaged cells into the supernatant. The amount of dead or plasma membrane-damaged cells is reflected by the amount of LDH release in the culture supernatant. After 3 h of OGD and 24 h of reperfusion, the cell medium supernatant was collected and incubated with the reaction mixture provided in the LDH activity assay kit (Nanjing Jiancheng Bioengineering Institute). The absorbance was measured at a wavelength of 450 nm using a microplate reader (Molecular Devices SpectraMax i3x).

### Immunofluorescence

Coverslips were placed in 6-well plates under normoxic conditions. The coverslips were washed three times with cold phosphate-buffered saline (PBS), fixed with 4% paraformaldehyde in PBS (pH 7.4) for 15 min at room temperature, and washed three times with PBS. The coverslips were then incubated for 15 min with 0.1% Triton X-100 at room temperature followed by three washes with PBS. Then, 10% serum from the secondary antibody species was used to block the samples for 30 min at 37 °C followed by incubation with primary antibodies (GFAP, 1:200, Cell Signaling Technology, #12389 and #3670; IBA-1, 1:100, Novus, #NBP2-19019; NLRP3, 1:100, Cell Signaling Technology, #15101; caspase-1, 1:100, Novus, #NB-100-56565) overnight at 4 °C; cells were then washed three times with PBS. Then, fluorescent secondary antibodies (1:100, Proteintech, #SA00013-1, SA00013-2, SA00013-3, and SA00013-4) were added for 1 h. After three washes with PBS, 1 μg/ml DAPI (Cell Signaling Technology, #4083) solution was added to cover the cells for 5 min in the dark followed by three washes with PBS. Fluorescence images were captured using a confocal laser-scanning microscope (Nikon A1 Ti, 400× or 600× magnification).

### Flow cytometry

Astrocyte apoptosis was measured by flow cytometry using a fluorescein isothiocyanate (FITC)-Annexin V/propidium iodide (PI) apoptosis detection kit (BD Biosciences, San Jose, CA, USA). Primary astrocytes cultured in 6-well plates were digested with 0.25% EDTA trypsin (Gibco). Cell precipitates were collected after centrifugation (1000 × g, 3 min) and washed with cold PBS. For Annexin V/PI analysis, cells were harvested and stained with 5 μl Annexin V and PI according to the manufacturer's instructions. The proportion of apoptotic cells was detected using a flow cytometer (BD Biosciences).

### Western blotting analysis

Astrocytes were lysed with RIPA buffer at 4°C for 30 min. Lysates were centrifuged at 14,000 × g for 20 min at 4 °C. The supernatant was collected, and the protein concentration was measured using a BCA Protein Assay kit (Thermo Fisher Scientific). Each sample (30-40 μg) was loaded onto 12% gels, electrophoresed, and transferred onto polyvinylidene difluoride (PVDF) membranes (Millipore, Billerica, MA, USA, #IPVH00010) at 4 °C. Non-fat milk (10%) was used to block the PVDF membranes for 1 h at room temperature. After blocking, the membranes were incubated with specific primary antibodies (caspase-12, 1:1000, Cell Signaling Technology, #2022; caspase-3, 1:1000, Abcam, #ab214430; NLRP3, 1:1000, Cell Signaling Technology, #15101; caspase-1, 1:1000, Novus, #NB-100-56565; IL-1β, 1:2000, Abcam, #ab9722; β-actin, 1:2000, Proteintech, #60008-1-Ig) overnight at 4 °C, followed by secondary antibodies (horseradish peroxidase-conjugated goat anti-rabbit or anti-mouse IgG) for 1 h at room temperature. Western blots were visualized using the Bio-Rad ChemiDoc^TM^ MP imaging system.

### Statistics analysis

All experiments were performed at least in triplicate. The experimental data were analyzed by one-way ANOVA with post-hoc tests (LSD test and S-N-K test) using SPSS 23.0 software. Data are presented as means ± standard deviation (SD). The level of statistical significance was defined as *P* < 0.05.

## Results

### The NLRP3-inflammasome was expressed in primary mouse astrocytes

**Figure 1:**
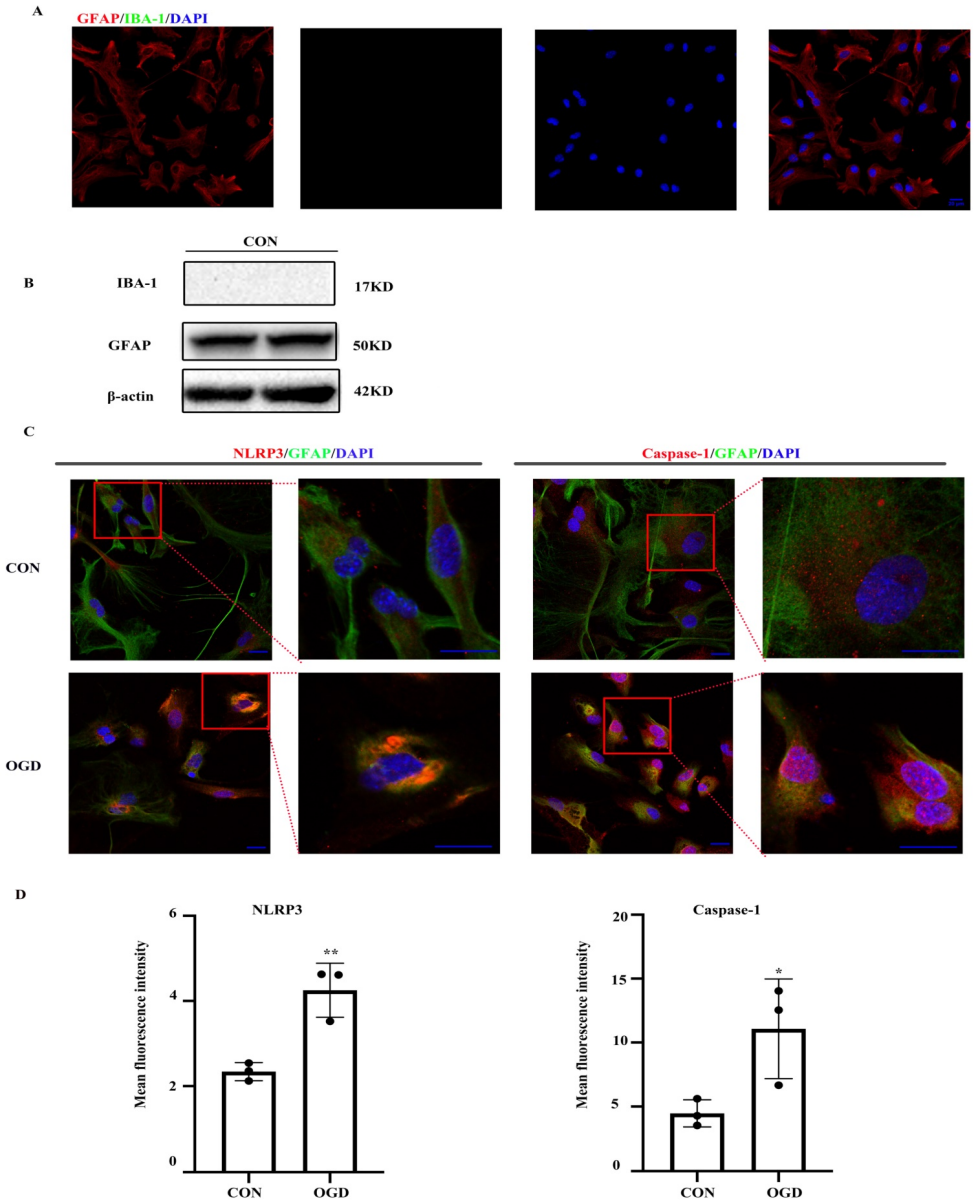
** NLRP3-inflammasome was expressed in primary mouse astrocytes.** NLRP3-inflammasome components were existed in primary mouse astrocytes. (**A,B**)The purity of primary mice astrocytes was detected by immunofluorescence and western blot (scale bar 20 µm, 400× magnification). (**C**) Immunofluorescence and was used to demonstrate the expression of NLRP3 and caspase-1 in primary mouse astrocytes in CON (control) group and OGD (3h hypoxia and 24h reperfusion) group (scale bar 20 µm, 600× or 1800x magnification). (**D**) Mean fluorescence intensity of NLRP3 and caspase-1 (comparisons to control group). Bar graphs were presented as means ± SD.*p<0.05, **p<0.01 (n=3).

The NLRP3 inflammasome, which consists of NLRP3, the apoptosis-associated speck-like protein containing a caspase-activating recruitment domain adaptor, and procaspase-1, has been linked to a number of diseases including ischemic stroke. First, we ensured the purity of primary mouse astrocytes (> 95%) (Fig. 1A, B). We then determined the expression of NLRP3 inflammasome components by immunofluorescence. Both NLRP3 and caspase-1 expression was observed in primary astrocytes under normal and OGD conditions (Fig. 1C). As shown in Figure 1C and D, NLRP3 and caspase-1 expression was upregulated following OGD.

### OGD induced cell injury and apoptosis in primary mouse astrocytes

**Figure 2:**
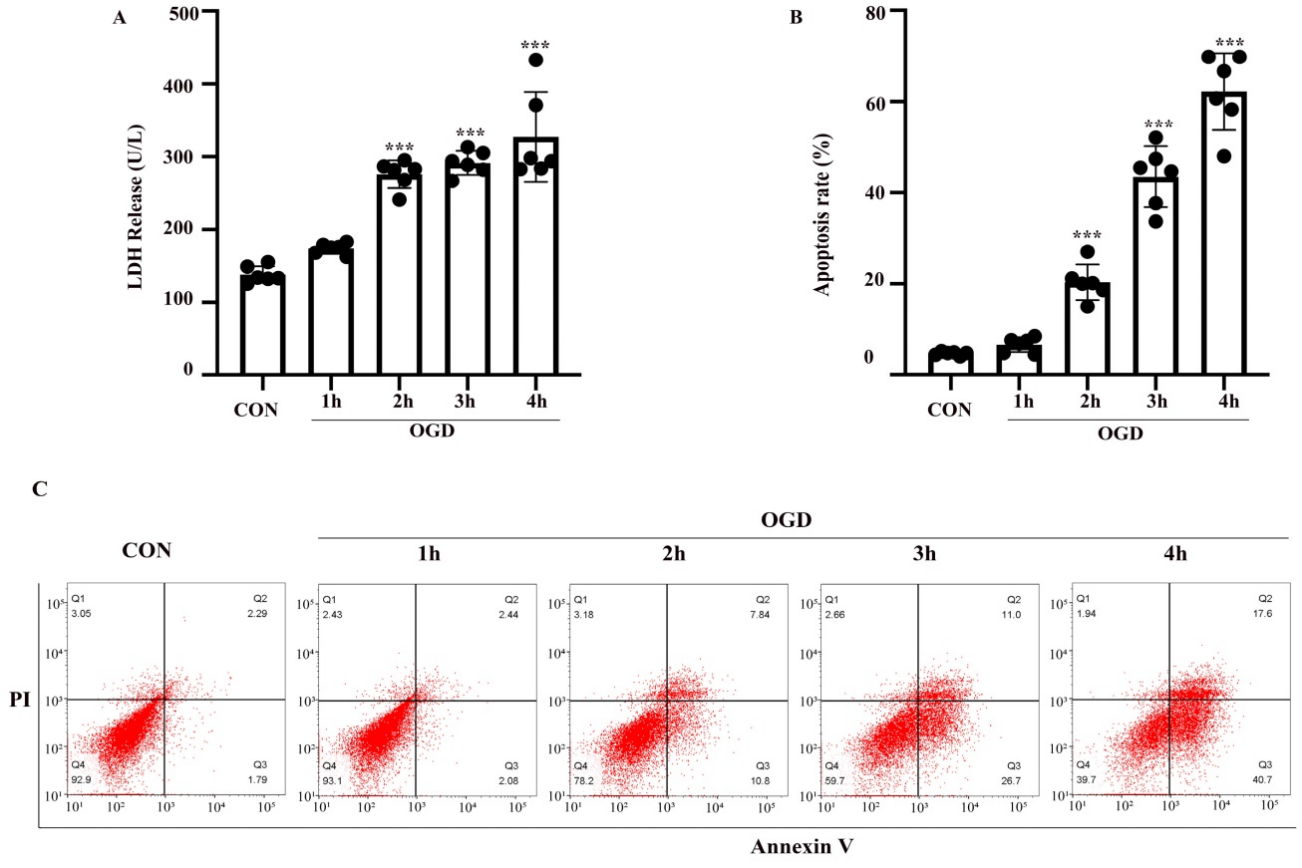
** OGD induced cell injury and apoptosis in primary mouse astrocytes.** Astrocytes were exposed to OGD for 1 to 4 hours and then returned to normal culture condition for 24 hours. (**A**) Cell injury was detected by LDH release assay. (**B**) Cell apoptosis was detected by Annexin V- fluorescein isothiocyanate (FITC)/prodium iodide (PI) flow cytometry, and the apoptosis rate was presented as the percentage of apoptotic cells vs total cells. (**C**) Representative scatterplots of Annexin V-FITC/PI flow cytometry in each group. CON: control group; OGD: Oxygen Glucose Deprivation group. *P < 0.05, **P<0.01, ***P<0.001(all comparisons to control group). Bar graphs were presented as means ± SD. (n=6),

The OGD model of astrocytes was established as described above. LDH and FITC-Annexin V/PI flow cytometry assays were performed to evaluate astrocyte injury and apoptosis. Astrocytes were exposed to 1-4 h of OGD and returned to normal conditions for 24 h. Compared with the control group, the rate of injury in astrocytes treated with OGD for 2-4 h was increased (*P* < 0.05) (Fig. 2A). Similarly, flow cytometry showed that apoptosis was increased after exposure to 2-4 h of OGD (Fig. 2B, C). The difference between the results shown in Fig. 2A and B might be due to the different properties of the two kits. The LDH release cytotoxicity assay measures membrane integrity, while flow cytometry was used to determine the rate of apoptosis. Therefore, astrocytes exposed to OGD for 3 h followed by 24 h of reoxygenation were used as the model for subsequent experiments.

### OGD increased the expression of NLRP3 inflammasome components, including NLRP3 and caspase-1, as well as caspase-12 and IL-1β expression in primary mouse astrocytes

**Figure 3:**
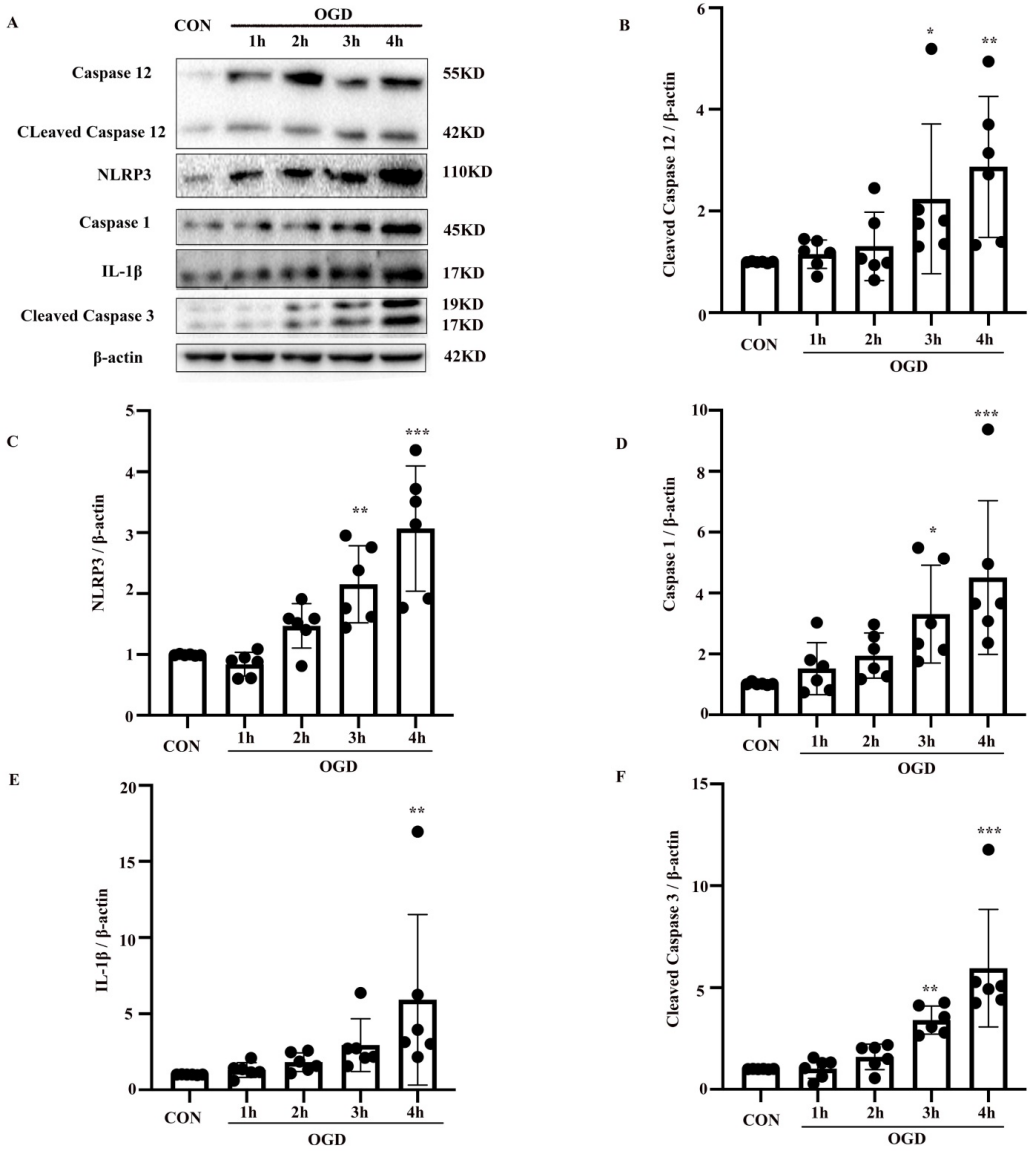
** OGD increased the expression of NLRP3 inflammasome components, including NLRP3 and caspase-1, as well as caspase-12 and IL-1β expression in primary mouse astrocytes.** (**A**) Western blot analysis of NLRP3, caspase-1, IL-1β, caspase-12 and cleaved caspase-3 in astrocytes exposed to 1, 2, 3, 4 hours of OGD and then returned to normal condition for 24 hours. β-actin was used as a loading control. Protein levels of cleaved caspase-12 (**B**) NLRP3 (**C**), caspase-1 (**D**), IL-1β (**E**), and cleaved caspase-3 (**F**) were normalized to β-actin and quantified by Image Lab software. CON: control group; OGD: Oxygen Glucose Deprivation group. *P < 0.05, **P<0.01, ***P<0.001(all comparisons to control group). Bar graphs were presented as means ± SD (n=6).

To confirm the involvement of the NLRP3 inflammasome and caspase-12 in OGD-induced astrocyte apoptosis, NLRP3, caspase-1, IL-1β, and caspase-12, as well as the apoptotic biomarker cleaved caspase-3, were analyzed in primary mouse astrocytes subjected to OGD. Compared with the control group, the protein levels of cleaved caspase-3 and caspase-12 (*P* < 0.05) were elevated in the OGD model (*P* < 0.05) (Fig. 3A-F). IL-1β and NLRP3 inflammasome components including NLRP3 and caspase-1 were also increased in the OGD groups compared with the control group (*P* < 0.05). These results suggest that OGD increases caspase-12 and IL-1β expression and NLRP3 inflammasome activation.

### Inhibition of caspase-12 prevented OGD-induced cell injury in primary mouse astrocytes

**Figure 4:**
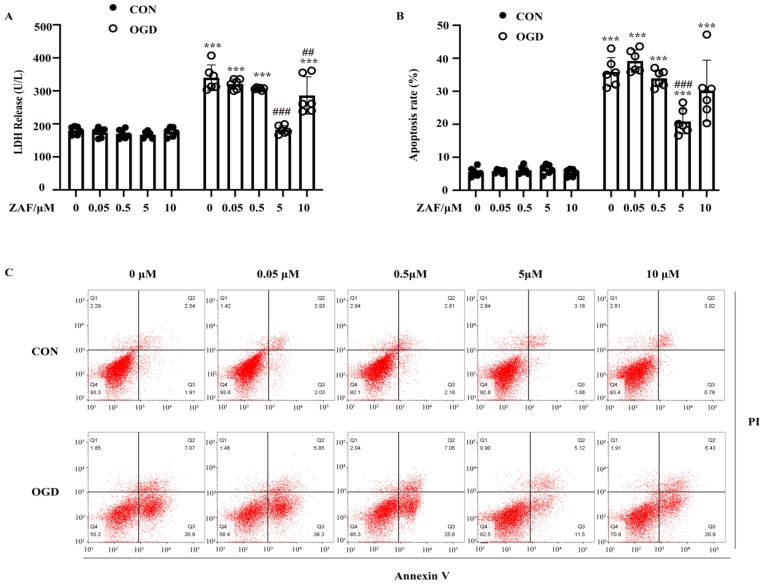
** Inhibition of caspase-12 prevented OGD-induced cell injury in primary mouse astrocytes.** Astrocytes were exposed to 3 hours of OGD and then 24 hours of reperfusion with or without ZAF (0, 0.05, 0.5, 5, 10 µM). (**A**) Cell injury was detected by LDH release assay. (**B**) Cell apoptosis was detected by Annexin V- fluorescein isothiocyanate (FITC)/prodium iodide (PI) flow cytometry, and the apoptosis rate was presented as the percentage of apoptotic cells vs total cells. (**C**) Representative scatterplots of Annexin V-FITC/PI flow cytometry in each group. CON: control group; OGD: Oxygen Glucose Deprivation group. *P < 0.05, **P<0.01, ***P<0.001 (all comparisons to control (ZAF=0μM) group); # *P* < 0.05,* ## P<0.01, ### P<0.001*(all comparisons to OGD(ZAF=0μM) group). Bar graphs were presented as means ± SD (n=6).

To verify the role of caspase-12 in OGD-induced cell injury in primary mouse astrocytes, we pretreated astrocytes with 0.05, 0.5, 5, or 10 μM ZAF. As shown in Figure 4A and B, compared with the OGD (0 μM ZAF) group, cell injury and apoptosis were significantly reduced by ZAF in a dose-dependent manner (*P* < 0.05). Taken together, these data demonstrate that inhibition of caspase-12 prevented OGD-induced cell injury in primary mouse astrocytes.

### Inhibition of caspase-12 attenuated NLRP3-inflammasome activation in OGD in primary mouse astrocytes

**Figure 5:**
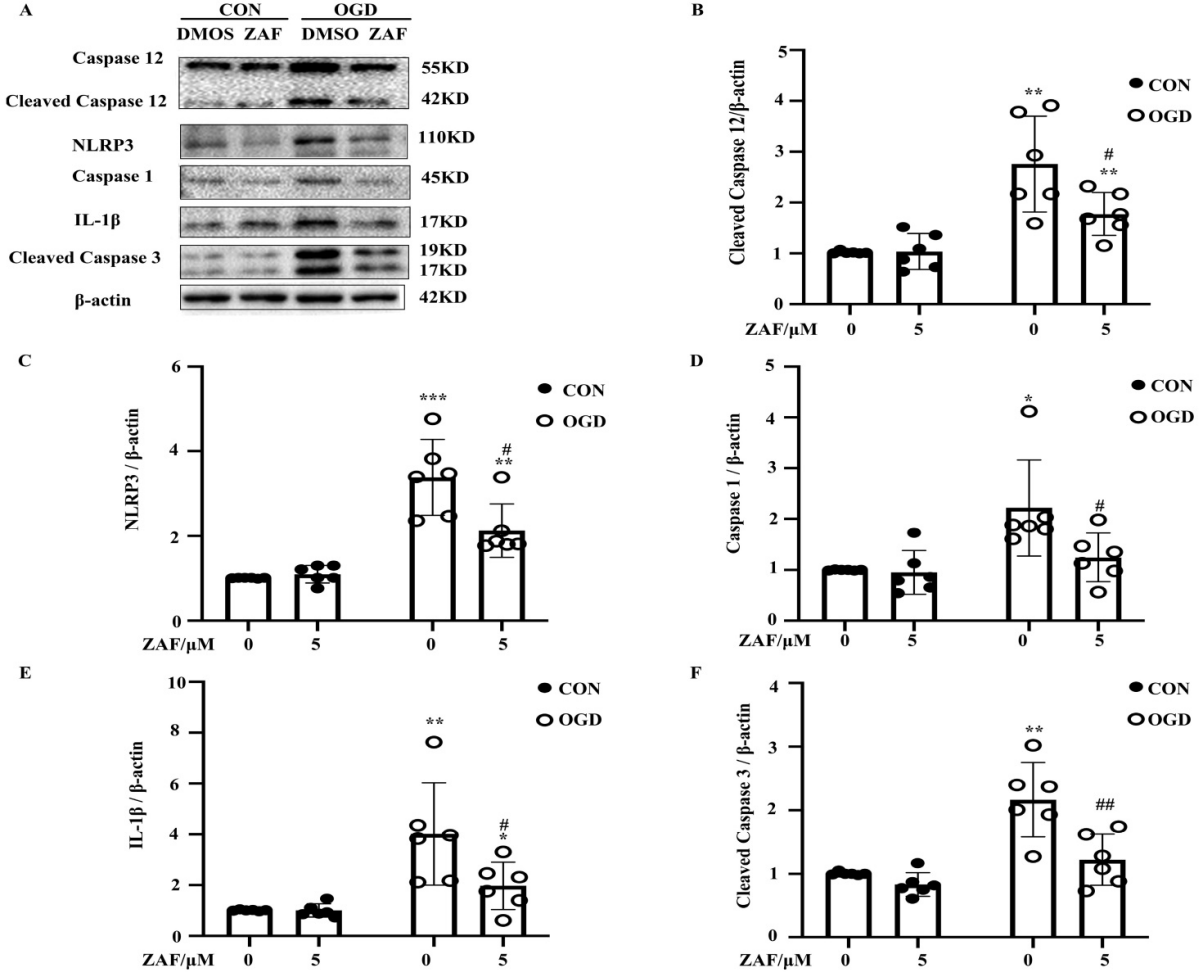
** Inhibition of caspase-12 attenuates NLRP3-inflammasome activation in OGD in primary mouse astrocytes.** (**A**) Western blot analysis of NLRP3, caspase-1, IL-1β, caspase-12 and cleaved caspase-3 in astrocytes exposed to 3 hours of OGD followed by 24 hours reperfusion with or without ZAF (5 µM). β-actin was used as a loading control. Protein levels of cleaved caspase-12 (**B**) NLRP3 (**C**), caspase-1 (**D**), IL-1β (**E**), and cleaved caspase-3 (**F**) were normalized to β-actin and quantified by Image Lab software. CON: control group; OGD: Oxygen Glucose Deprivation group. *P < 0.05, **P<0.01, ***P<0.001 (all comparisons to control (ZAF=0 µM) group); # *P* < 0.05,* ## P<0.01, ### P<0.001* (all comparisons to OGD (ZAF=0 µM) group). Bar graphs were presented as means ± SD (n=6).

As shown in Figure 5, activation of caspase-12 by OGD was significantly downregulated in primary mouse astrocytes when pre-incubated with ZAF (5 μM) (*P* < 0.05) (Fig. 5A, B). Cells pre-incubated with ZAF (5 μM) did not exhibit any significant changes in NLRP3 inflammasome components including NLRP3, caspase-1, and IL-1β as well as cleaved caspase-3 in the control group (*P* > 0.05) (Fig. 5). In addition, ZAF (5 μM) dramatically attenuated the upregulation of NLRP3, caspase-1, IL-1β, and cleaved caspase-3 in the OGD groups (Fig. 5A-F) (*P* < 0.05). These results indicate that inhibition of caspase-12 was essential for attenuating activation of the NLRP3 inflammasome in primary mouse astrocytes subjected to OGD.

## Discussion

I/R injury elicits damage to neurons and non-neuronal cells. Reperfusion after ischemia triggers an inflammatory cascade, resulting in secondary brain injury after ischemia. Neurons are more vulnerable than astrocytes to I/R injury; thus, enhancing the survival of astrocytes provides neuroprotection against I/R injury 21. Therefore, a better understanding of the potential mechanisms of astrocytes against I/R damage in stroke would help prevent further neuronal dysfunction. Astrocytes are the most abundant non-neuronal cells in the brain. Studies have suggested that astrocytes participate in regulation of innate immunity in the CNS. Astrocytes can affect the physiological status of neurons and may exacerbate neuronal damage during I/R injury and other types of brain damage 22-24 . The astrocytic response to stroke is complex and incompletely understood 25. Following ischemic stroke, astrocytes perform both deleterious and beneficial functions. Considering the role of astrocytes in cerebral ischemic injury, targeting this cell type as a novel therapeutic strategy in ischemic stroke is worth exploring.

Evidence has demonstrated that ERS results in NLRP3 inflammasome activation 26, and in turn the NLRP3 inflammasome might also cause ERS, although the underlying mechanisms remain unclear 27 . Caspase-12 is predominantly localized in the ER and is especially activated by ERS 12, 28. Caspase-12 has also been reported to activate the downstream molecule caspase-3, a traditional pro-apoptotic protein, in ERS-induced neuronal apoptosis 29. In addition to its role in apoptosis, caspase-12 generally acts as a regulator of the inflammatory response, which has been attributed to its relationship with inflammasome complexes 30. Caspase-12 is an inflammatory caspase that has been reported to negatively regulate inflammation 31, 32, although one study questioned its role in inflammasome activation 14.

Cleaved caspase-12 up-regulation has been observed after I/R injury but its roles in apoptosis are controversial 33, 34. Our results indicate that the levels of cleaved caspase-12 and cleaved caspase-3 as well as rate of apoptosis were elevated following I/R injury in primary mouse astrocytes. To evaluate the role of caspase-12 in I/R injury, we evaluated the effects of ZAF. When astrocytes were pretreated with ZAF, both the proportion of apoptotic cells and level of cleaved caspase-3 were significantly lower compared with the untreated groups, providing strong evidence that caspase-12 plays an essential role in inducing injury in astrocytes after OGD.

The inflammatory response is important in the pathophysiology of ischemic stroke. Several potential molecules are implicated in cerebral ischemic inflammation, and essential functions of inflammasomes have been identified. Recent studies showed that various inflammasomes, containing NLRP1, NLRP2, NLRP3, NLRP10, NLR family card domain containing 4 (NLRC4), and absent in melanoma 2(AIM2) 35-39, are activated in ischemic stroke. Notably, evidence indicated that the NLRP2 inflammasome is also expressed in astrocytes 40. The NLRP3 inflammasome in particular has been widely explored in brain ischemia because of its vital contributions to neuronal death and behavioral disorders following stroke 41. Evidence has suggested that inhibition of NLRP3 activation attenuates infarction volumes and ameliorates stroke outcomes 42. However, most of the current inflammasome studies of CNS disorders have focused on microglia. Gustin et al. suggested that the NLRP3 inflammasome is limited to mouse microglia but not astrocytes 17. Nevertheless, increasing evidence supports the presence of the NLRP3 inflammasome in other brains cells, such as oligodendrocytes and astrocytes, in pathological conditions and some disease models 18, 43. Primary astrocytes were purified of microglia in this study, and the existence of the NLRP3 inflammasome in primary mouse astrocytes was demonstrated (Fig. 1C, D). Interestingly, the expression of NLRP3, caspase-1, and IL-1β was attenuated by ZAF, which to our knowledge is the first report of the effect of ZAF on NLRP3 inflammasome activation. Inflammasomes are cytosolic protein complexes involved in the innate immune system. OGD treatment of primary mouse astrocytes stimulated NLRP3 inflammasome activation and thus upregulation of procaspase-1 and IL-1β expression, consistent with a previous study 44. IL-1β is primarily an anti-inflammatory cytokine that contributes to I/R cerebral damage and CNS inflammatory responses 45. The generation of IL-1β is mediated by inflammasomes and the protease caspase-1 46. As shown in the present study, ZAF could decrease the generation of IL-1β and thus alleviate astrocyte I/R injury.

Activation of caspase-12 is closely related to brain ischemia. Inhibition of ERS-induced caspase-12 activation potentially decreases cerebral damage in stroke and thereby damage-associated molecular pattern-induced NLRP3 inflammasome activation. However, there are several questions remain unanswered. First, the effect of ZAF *in vivo* will be addressed in future work. Second, the reciprocal effect of the NLRP3 inflammasome on caspase-12 is worth exploring. Finally, it would be interesting to investigate whether the pyroptosis pathway is involved in our model.

## Conclusion

Taken together, we demonstrated the existence of the NLRP3 inflammasome in primary mouse astrocytes. ZAF inhibited caspase-12-mediated primary mouse astrocyte apoptosis following I/R injury, and ZAF may be involved in alleviation of NLRP3 inflammasome activation. This study suggests that caspase-12 and its potential regulation of NLRP3 inflammasome activation may be a promising target for treatment of ischemic stroke.

## Abbreviations

OGD: oxygen-glucose deprivation
ZAF: caspase-12 specific inhibitor Z-ATAD-FMK
CNS: central nervous system
ERS: endoplasmic reticulum stress
I/R: ischemia/reperfusion
NLRP3: nucleotidebinding domain leucine-rich repeat containing receptors pyrin domain containing 3
LDH: Lactate dehydrogenase
PVDF: polyvinylidenedifluoride
ASC: apoptosis-associated speck-like protein containing a caspase-activating recruitment domain
NLRC4: NLR family card domain containing 4
AIM2: absent in melanoma 2
DAMPs: damage-associated molecular patterns
